# Association between body roundness index and overactive bladder: results from the NHANES 2005–2018

**DOI:** 10.1186/s12944-024-02174-1

**Published:** 2024-06-12

**Authors:** Yujun Zhang, Jingjing Song, Benjie Li, Yunhao Wu, Shengjun Jia, Hongxin Shu, Fang Liu, Xiaorong Yang

**Affiliations:** 1 Department of Urology Surgery, The First Affiliated Hospital, Jiangxi Medical College, Nanchang University, 17 Yongwai Zheng Street, Nanchang, Jiangxi 330006 China; 2 Huankui Academy, Nanchang University, Nanchang, Jiangxi 330006 China; 3 School of Ophthalmology, Optometry of Nanchang University, Nanchang, Jiangxi 330006 China; 4 Queen Mary School, Nanchang University, Nanchang, Jiangxi 330006 China; 5 The Second Clinical Medical College, Nanchang University, Nanchang, Jiangxi 330006 China; 6 Department of Neurosurgery, The Second Affiliated Hospital of Nanchang University, Nanchang, Jiangxi 330006 China; 7 Department of Health Management Medicine, The First Affiliated Hospital, Jiangxi Medical College, Nanchang University, 17 Yongwai Zheng Street, Nanchang, Jiangxi 330006 China; 8 Department of Urology Surgery, The First Hospital of Nanchang, Nanchang, Jiangxi 330008 China; 9 Department of Urology Surgery, The People’s Hospital of Jing An County, Yichun, Jiangxi 330600 China

**Keywords:** Body roundness index, Overactive bladder, Cross-sectional study, NHANES

## Abstract

**Background and aim:**

An increasing amount of research has indicated obesity greatly affects individuals with overactive bladder (OAB). However, traditional anthropometric methods present challenges in accurately assessing the likelihood of OAB. Hence, this study’s objective was to identify the correlation between the body roundness index (BRI) and OAB.

**Methods:**

The research included 12,401 individuals who participated in the National Health and Nutrition Examination Survey spanning 2005–2018. The correlation between BRI and OAB was explored by using weighted multiple logistic regression and weighted restricted cubic spline (RCS). Subgroup analyses showed the associations based on different population types. The study also analyzed the predictive capability of various anthropometric indices, including BRI, body mass index, waist circumference, and weight, in assessing the likelihood of OAB through Receiver-operating characteristic (ROC) curves.

**Results:**

An independent positive correlation between OAB and BRI was identified after adjusting for potential confounders in weighted multivariate logistic models[odds ratio (OR) = 1.15, 95% confidence interval (CI), 1.12–1.17]. Weighted RCS analysis found a positive dose-response correlation between OAB and BRI. The effect size of BRI on OAB remained stable across all prespecified subgroups (all *P* for interactions > 0.05). In ROC analysis, BRI showed better discriminatory ability for OAB compared with other anthropometric measures for both genders (all *P* < 0.01). The best BRI cutoff for predicting OAB was lower for men (5.151) than for women (5.383), suggesting that men were more susceptible to changes in BRI than women.

**Conclusions:**

This study demonstrated that a raised BRI is correlated with a higher likelihood of OAB. Due to the effectiveness and non-invasiveness of BRI in predicting OAB, it is expected to become the preferred method for early detection and management strategies.

**Supplementary Information:**

The online version contains supplementary material available at 10.1186/s12944-024-02174-1.

## Introduction

Overactive bladder (OAB) is a urologic disease characterized by the urgency of urination, often with nocturia and frequent urination, but with no urinary tract infection and other apparent pathologic abnormalities [1]. The prevalence of OAB varies among countries or regions, and it may be related to ethnicity, lifestyle habits, and data collection standards. Over the past four decades, the prevalence of OAB in European countries has been 13.4% and 14.6% for males and females, respectively [2], while an epidemiological study in China revealed a total prevalence of around 6.0% [3]. As predicted by Lrwin et al., the global prevalence of OAB might reach 20% in 2018 [4]. OAB can affect patients’ quality of life to different degrees. The urinary urgency symptoms significantly impact patients’ work efficiency and interfere with their social functioning [5]. According to statistics, OAB results in annual medical expenses of billions of dollars in the United States [6].

Increasing research indicates that obesity significantly increases the risk of OAB [7, 8]. The exact mechanisms linking obesity, overweight, and OAB remain unclear. Numerous scientists now acknowledge that being overweight can elevate intra-abdominal pressure, subsequently raising bladder and intravesical pressure, ultimately contributing to OAB [9]. OAB can be predicted by longer waist circumference (WC), increased fasting blood glucose levels, elevated levels of low-density lipoprotein, and a higher body mass index (BMI) [10]. Women whose BMI is 30 or above are at a higher risk of nocturnal urinary urgency than those with a normal BMI [11]. Furthermore, for every 5-unit rise in BMI, the likelihood of OAB may increase by 20–70% [12].

Recently, researchers have paid more attention to understanding the relationship between OAB and visceral adiposity. A study by Hashimoto et al. identified a positive association between the excessive build-up of visceral fat and the risk of OAB in women [13]. Traditional measures like BMI and WC are limited in their accuracy for predicting OAB risk. Focal distribution of fat in the bladder wall and a higher surface area of visceral fat are emerging risk factors for developing OAB [14]. Thomas et al. developed the body roundness index (BRI) to offer a relatively accurate measurement to assess the volume of body fat and visceral adiposity [15]. BRI provides a more accurate assessment of body fat percentage and visceral fat than the conventional body composition index, making it superior to BMI and WC [15]. BRI is also associated with other urological conditions, such as erectile dysfunction [16] and kidney stones [17]. The relationship between OAB and BRI has not been explored in any studies to date. By identifying individuals at risk for OAB based on their BRI, healthcare professionals can implement targeted interventions and preventive measures at an early stage. This, in turn, has the potential to improve patient outcomes, enhance quality of life, and reduce the burden on healthcare resources. Hence, this research aimed to fill this knowledge gap by conducting a cross-sectional analysis of a representative American population using data from the National Health and Nutrition Examination Survey (NHANES) database. Additionally, this study explored the feasibility of BRI as a predictive tool of OAB in order to offer valuable insights for early intervention and management of OAB.

### Study participants and study design

NHANES is a research project based on the U.S. population that investigates the wellness and nutritional well-being of Americans, along with performing epidemiological research. Individuals participating in this study all provided their consent in writing, and all the data can be accessed directly on an online website (https://www.cdc.gov/nchs/nhanes/index.htm).

### Outcome variable: OAB

As defined by the International Continence Society, UUI and nocturia are the main features of OAB, and their evaluation constitutes the assessment of OAB. The survey was conducted by trained researchers using questionnaires. To evaluate the degree of UUI, participants were asked two questions: [1] “During the last 12 months, have you leaked or lost control of even a small amount of urine with an urge or pressure to urinate and you could not get to the toilet fast enough?” [2] “How frequently does this occur?” Nocturia was evaluated by asking another question: “During the last 30 days, how many times per night did you most typically get up to urinate, from the time you went to bed at night until the time you got up in the morning?”. After collecting the results, the researchers employed the Overactive Bladder Symptom Score (OABSS) to determine the severity of OAB [18]. Participants scoring 3 or more were grouped as having OAB [19–21]. All details are outlined in Supplementary Fig. 1.

### Anthropometric measures

WC was gauged in centimeters utilizing a tape measure at the superior border of the iliac crest. BMI and BRI were determined using previously published formulas: [1] BMI = weight/height^2^ [2]. BRI = 364.2-365.5*(1-[WC(m)/2π]^2^ /[0.5*height(m)]^2^ ) ^½^ [15].

### Covariates

This study incorporated some possible covariates that could affect the correlation between BRI and OAB according to prior research and clinical considerations [22–25]. Age, total cholesterol (TC), low-density lipoprotein cholesterol (LDL-C), fasting blood glucose (FBG), and triglycerides (TG) were all considered continuous variables. The categorical variables included gender (female and male), race (non-Hispanic white, non-Hispanic black, Mexican American, other races), educational attainment (high school and less, more than high school), marital status (married or living with a partner, living alone), family poverty income ratio (PIR) [ low (≤ 1), middle (> 1 and < 4), and high (≥ 4)] [26], smoking status (never, former, and now), drinking status (never, mild, moderate, heavy, former) [27], hypertension (no/yes), and diabetes (no/yes). The detailed measurement procedures are available for public access on the website https://www.cdc.gov/nchs/nhanes.

### Statistical analysis

To enhance the precision of the data and thus minimize the impact of the intricate multi-stage sampling design employed in NHANES, the research applied sample weights as recommended by the NHANES guidelines. The categorical variables are represented as weighted percentages, while the continuous variables as weighted means and standard errors. To investigate the correlation between BRI and OAB, weighted multivariate logistic regressions were conducted, adjusting the continuous or quantile outcomes for different covariates. Model 1 is unadjusted. Model 2 controlled for race, age, gender, educational attainment, and marital status. Model 3 controlled for PIR, race, age, gender, educational attainment, marital status, smoking status, drinking status, hypertension, diabetes, FBG, TC, TG, and LDL-C. Additionally, weighted RCS curves were used in Model 3 to evaluate the association between BRI and OAB. Subgroup analyses were conducted according to age, gender, race, educational attainment PIR, smoking status, marital status, drinking status, hypertension, and diabetes. The discriminatory capacity of BRI, BMI, WC, and weight in predicting OAB was assessed by examining receiver-operating characteristic (ROC) curves and calculating the area under the curve (AUC). The optimal cut-off values of BRI, BMI, WC, and weight were derived using the maximum Youden index.

The statistical analysis was conducted using R version 4.2.2 (http://www.Rproject.org). A *P* value of less than 0.05 on both sides is considered to be a statistically significant difference.

## Results

### Baseline characteristics of participants

**Fig. 1:**
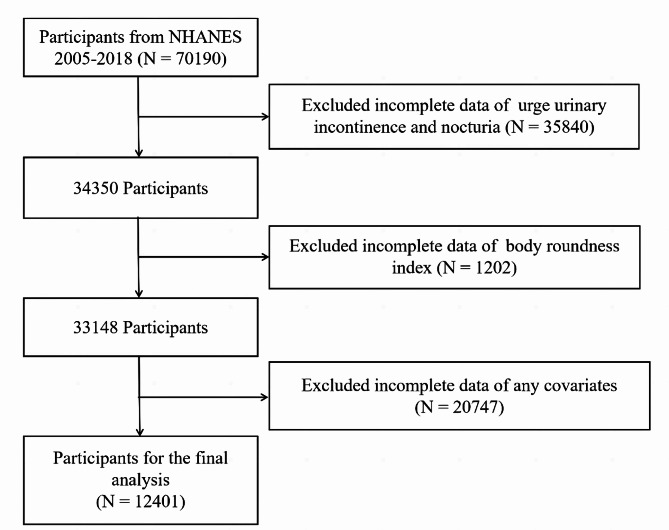
Flow diagram of study cohort selection

The research enrolled 70,190 individuals from NHANES 2005–2018. 35,840 participants were eliminated from the study because of incomplete data on urinary urgency incontinence (UUI) and nocturia. Moreover, a total of 1202 and 20,747 individuals were not included because of incomplete data regarding BRI and covariates. Ultimately, the final sample size for this study was 12,401 (Figs. 1), 2,441 of whom had OAB (Table 1). The input variables used to characterize the chosen dataset encompassed multiple factors, including age, gender, race, educational attainment, PIR, smoking status, marital status, drinking status, hypertension, diabetes, BMI (kg/m^2^), FBG (mg/dL), WC (m), TC (mg/dL), TG (mg/dL), height (m), LDL-C (mg/dL), weight (kg), and BRI. Non-Hispanic whites constituted the largest proportion in both OAB and non-OAB populations. Among all participants with a mean age of 47.03 ± 0.28 years, 6173 were men (49.24%) and 6228 were women (50.76%).

**Table 1:** Weighted characteristics of the study population^a^

Characteristics	Total	Non-overactive bladder	Overactive bladder	*P*-value
**Number**	12,401	9960	2441	
**Age (years)**	47.03 (0.28)	45.17 (0.27)	57.55 (0.44)	**< 0.001**
**Gender (%)**				**< 0.001**
Male	6173 (49.24)	5160 (51.38)	1013 (37.20)	
Female	6228 (50.76)	4800 (48.62)	1428 (62.80)	
**Race (%)**				**< 0.001**
Non-Hispanic White	5610 (69.50)	4586 (70.20)	1024 (65.55)	
Non-Hispanic Black	2436 (10.57)	1793 ( 9.56)	643 (16.28)	
Mexican American	1908 ( 7.97)	1529 (8.00)	379 (7.82)	
Other Race	2447 (11.95)	2052 (12.24)	395 (10.35)	
**Educational Attainment (%)**				**< 0.001**
High school or less	5697 (38.21)	4291 (35.99)	1406 (50.72)	
More than high school	6704 (61.79)	5669 (64.01)	1035 (49.28)	
**Marital Status (%)**				**< 0.001**
Married or living with partner	7549 (64.38)	6225 (65.26)	1324 (59.41)	
Living alone	4852 (35.62)	3735 (34.74)	1117 (40.59)	
**PIR (%)**				**< 0.001**
Low	3287 (36.78)	2835 (38.46)	452 (27.36)	
Middle	2471 (13.37)	1846 (12.35)	625 (19.13)	
High	6643 (49.85)	5279 (49.19)	1364 (53.51)	
**Smoking status (%)**				**0.006**
Never	6812 (54.77)	5594 (55.58)	1218 (50.24)	
Now	2497 (19.92)	1977 (19.53)	520 (22.10)	
Former	3092 (25.31)	2389 (24.89)	703 (27.66)	
**Drinking status (%)**				**< 0.001**
Never	1643 (10.37)	1231 ( 9.63)	412 (14.55)	
Mild	4363 (37.79)	3572 (38.12)	791 (35.95)	
Moderate	1901 (17.52)	1603 (18.19)	298 (13.72)	
Heavy	2507 (21.34)	2124 (22.44)	383 (15.18)	
Former	1987 (12.98)	1430 (11.62)	557 (20.61)	
**Hypertension (%)**				**< 0.001**
No	7204 (62.99)	6295 (66.60)	909 (42.66)	
Yes	5197 (37.01)	3665 (33.40)	1532 (57.34)	
**Diabetes (%)**				**< 0.001**
No	9881 (85.00)	8305 (87.46)	1576 (71.16)	
Yes	2520 (15.00)	1655 (12.54)	865 (28.84)	
**FBG (mg/dL)**	105.43 (0.38)	103.84 (0.37)	114.35 (1.00)	**< 0.001**
**TC (mg/dL)**	192.18 (0.55)	192.06 (0.58)	192.85 (1.20)	0.521
**TG (mg/dL)**	117.01 (0.97)	115.83 (1.06)	123.67 (1.61)	**< 0.001**
**LDL-C (mg/dL)**	114.24 (0.44)	114.40 (0.48)	113.37 (1.00)	0.347
**BMI (kg/m**^**2**^)	28.95 (0.10)	28.57 (0.11)	31.12 (0.22)	**< 0.001**
**Weight (kg)**	83.06 (0.29)	82.58 (0.30)	85.79 (0.66)	**< 0.001**
**WC (m)**	0.99 (0.00)	0.98 (0.00)	1.05 (0.00)	**< 0.001**
**Height (m)**	1.69 (0.00)	1.70 (0.00)	1.66 (0.00)	**< 0.001**
**BRI**	5.33 (0.04)	5.11 (0.04)	6.39 (0.07)	**< 0.001**

Abbreviations: PIR, family poverty income ratio; FBG, fasting blood glucose; TC, total cholesterol; TG, triglyceride; LDL-C, low-density lipoprotein cholesterol; BMI, body mass index; WC, waist circumstance; BRI: body roundness index

^a^Values are weighted means (standardized error) or number of participants (weighted percentages) unless otherwise indicated

### Correlation between OAB and BRI

**Fig. 2:**
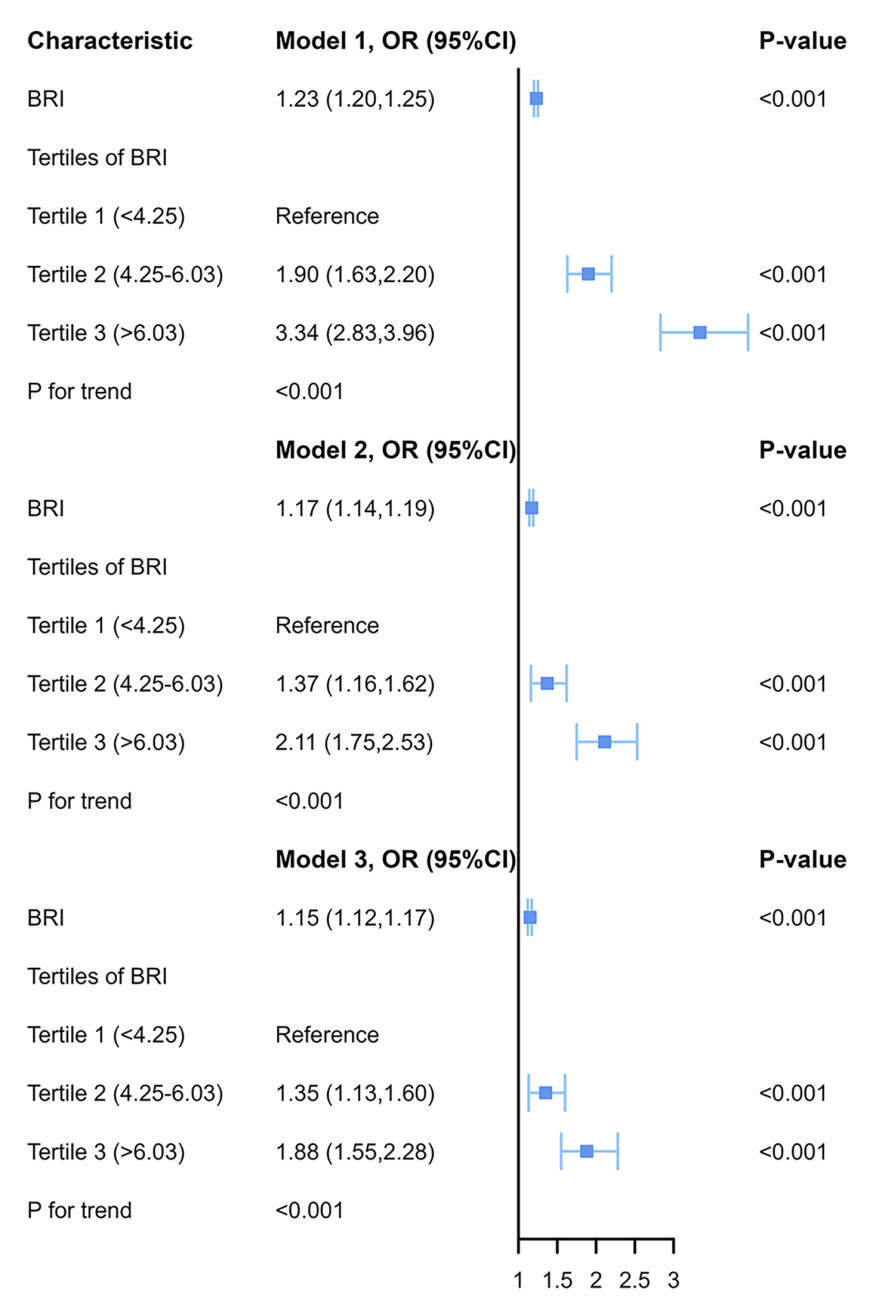
Weighted multivariate logistic regression analysis of BRI and overactive bladder

The relationship between BRI and OAB was investigated using weighted multivariate logistic regression models. As shown in Fig. 2, the BRI values in Models 1–3 were 1.23 (95% CI, 1.20–1.25), 1.17 (95% CI, 1.14–1.19), and 1.15 (95% CI, 1.12–1.17), respectively. In Model 1, a positive association was found between OAB and BRI within the subpopulation (OR: 1.90, 95% CI, 1.63–2.20 for Tertile 1 compared with Tertile 2; OR: 3.34, 95% CI, 2.83–3.96 for Tertile 1 compared with Tertile 3). This significance persisted in Model 2, which made adjustments for age, gender, race, educational attainment, and marital status (OR: 1.37, 95% CI, 1.16–1.62 for Tertile 1 compared with Tertile 2; OR: 2.11, 95% CI, 1.75–2.53 for Tertile 1 compared with Tertile 3). Finally, in Model 3, which included additional adjustments for PIR, smoking and drinking status, hypertension, diabetes, FBG, TC, TG, and LDL-C, the correlation between OAB and BRI remained significant in the subpopulation (OR: 1.35, 95% CI, 1.13–1.60 for Tertile 1 compared with Tertile 2; OR: 1.88, 95% CI, 1.55–2.28 for Tertile 1 compared with Tertile 3). The correlations between other anthropometric measurements and OAB were also investigated, including BMI, WC, and weight. Similar to BRI, these measurements were significantly associated with OAB (Supplementary Table 1).

**Table 2:** Diagnostic efficacy of ROC analysis of obesity-related indices for overactive bladder

Anthropometric Measures	Best thresholds	Sensitivity	Specificity	Positive predictive value	Negative predictive value	AUC (95%CI)	*P* for difference in AUC
BRI	5.124	0.444	0.670	0.270	0.873	0.652 (0.641–0.664)	Reference
BMI	30.185	0.335	0.492	0.265	0.842	0.601 (0.589–0.614)	**< 0.001**
WC	0.974	0.480	0.660	0.252	0.862	0.620 (0.608–0.632)	**< 0.001**
Weight	88.950	0.308	0.378	0.231	0.819	0.548 (0.535–0.560)	**< 0.001**

Abbreviations: PIR, family poverty income ratio; FBG, fasting blood glucose; TC, total cholesterol; TG, triglyceride; LDL-C, low-density lipoprotein cholesterol; BMI, body mass index; WC, waist circumstance; BRI: body roundness index

### Nonlinear correlation between OAB and BRI

**Fig. 3:**
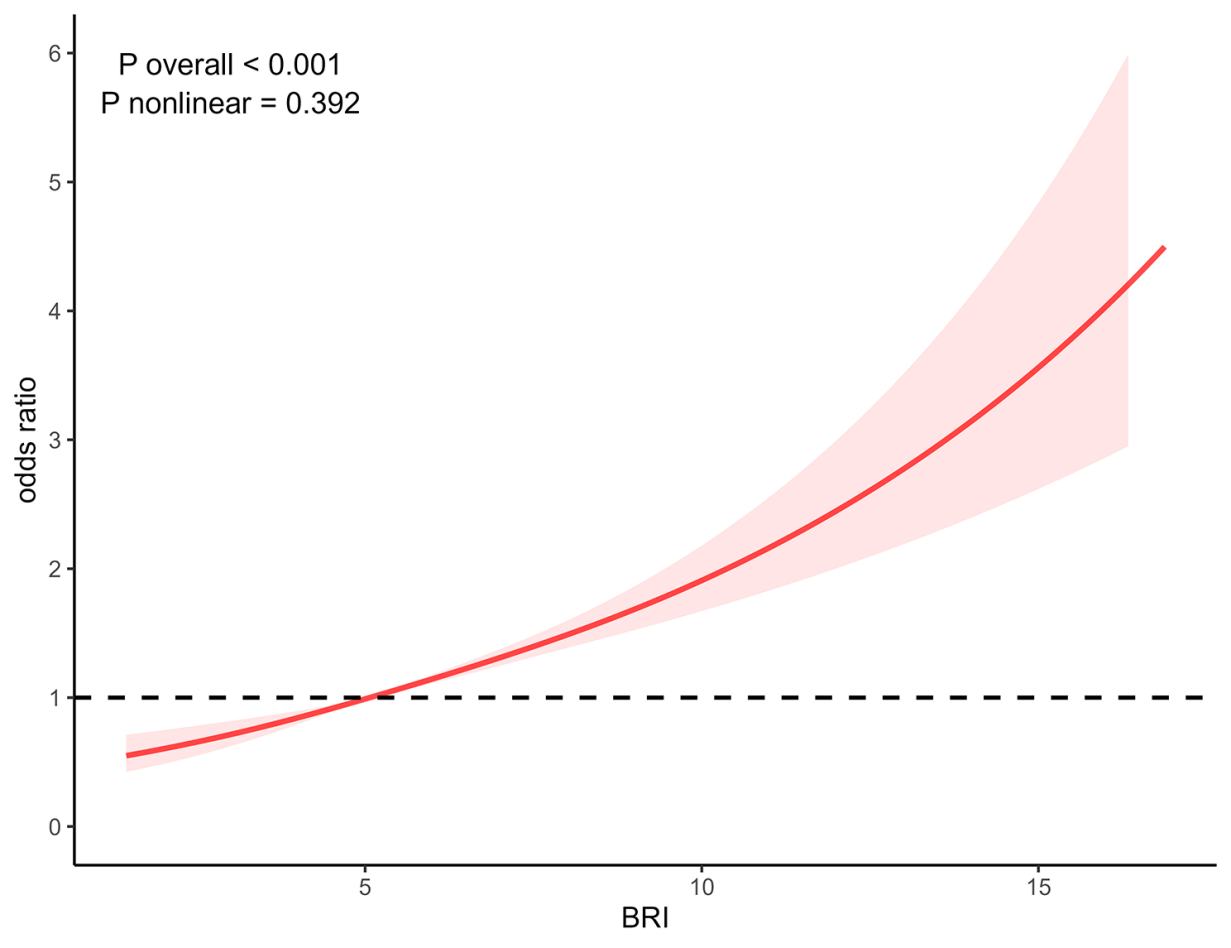
Nonlinear correlation between OAB and BRI

To further investigate and visualize any possible nonlinear correlation between OAB and BRI, a weighted multivariable–adjusted RCS analysis was conducted (Fig. 3). The findings indicated no nonlinear correlation between OAB and BRI, as the *P* value for nonlinearity was 0.392. There was a positive dose-response relationship between BRI and OAB (P overall < 0.001).

### Subgroup analyses

**Fig. 4:**
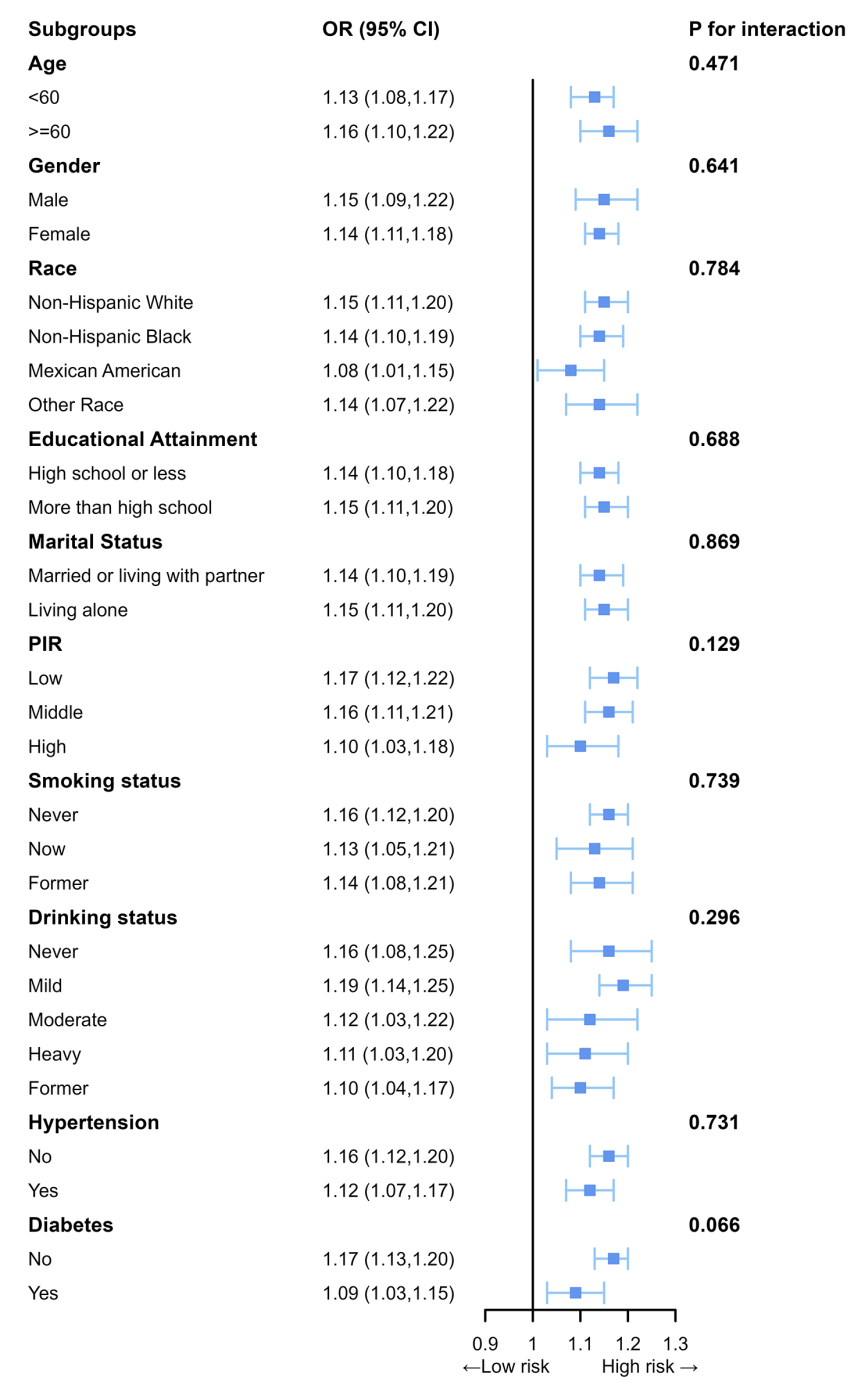
The association between OAB and BRI in various subgroups

Subgroup analyses were employed in this research to identify whether the relationship between OAB and BRI varied among different subgroups. The interaction test did not reveal any significant differences across other stratifications, indicating that the positive correlation between OAB and BRI remained strong and consistent across all subgroups (Fig. 4).

### BRI as a predictor for OAB

**Fig. 5:**
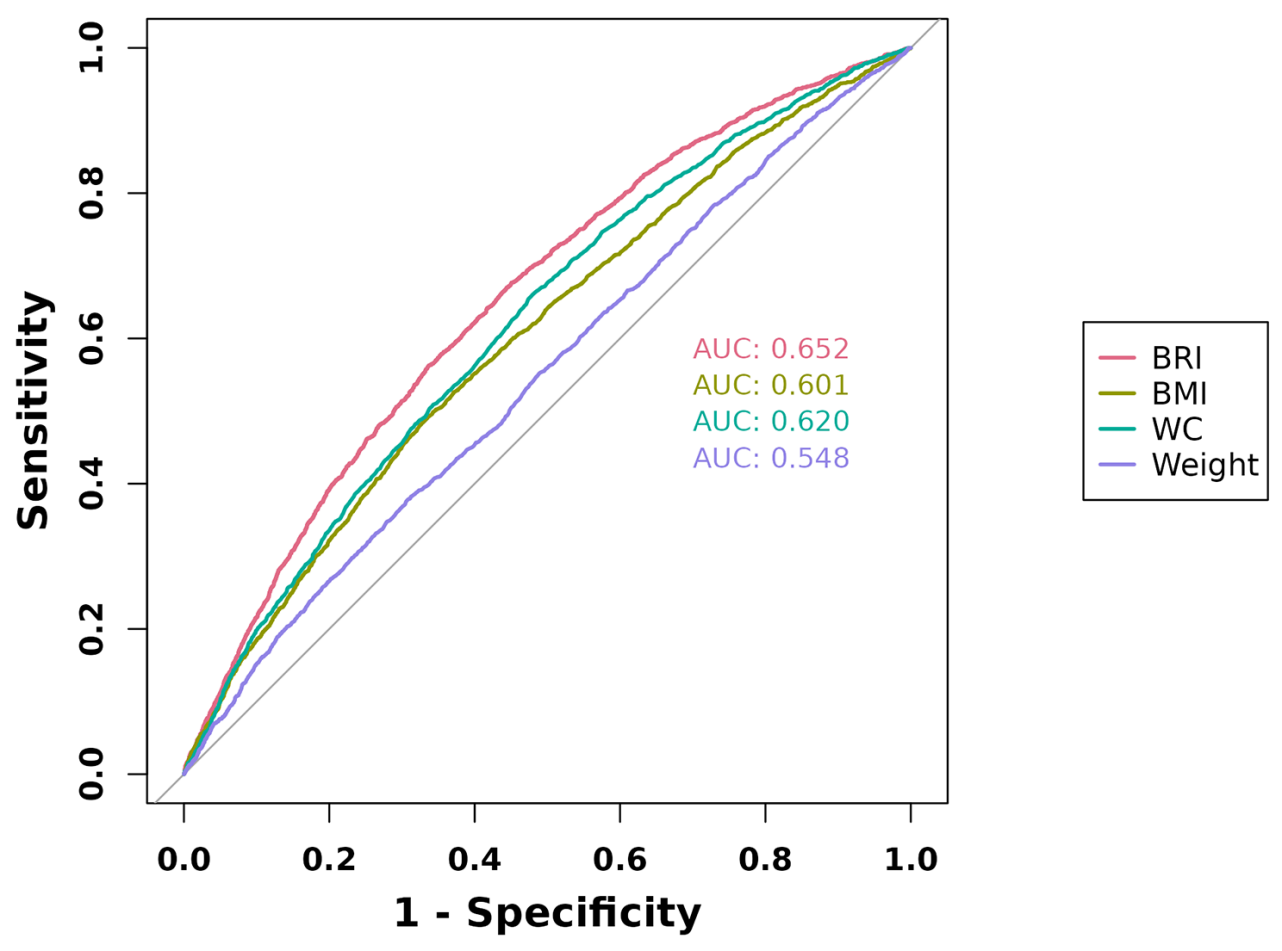
Receiver operating characteristic (ROC) curve analysis for predicting overactive bladder

Weight and BMI are the most widely utilized anthropometric indicators, whereas WC is a frequently employed measure to assess obesity. The AUC was calculated to compare the efficacy of the BRI and various anthropometric indicators in predicting the likelihood of OAB (Fig. 5). In the present analysis, the BRI demonstrated superior performance compared with all four anthropometric indices (AUC 0.652, 95% CI, 0.641–0.664, all *P* < 0.001). The optimum BMI cutoff value was determined to be 5.124 (**Table 2**). Analogously, in male and female subpopulations, BRI also demonstrated superior performance compared with all four anthropometric indices (AUC 0.626, 95% CI, 0.608–0.645 for males; AUC 0.660, 95% CI, 0.645–0.676 for females; all *P* < 0.001) (Supplementary Figs. 2–3). Intriguingly, this research’s findings suggested that the optimal threshold value of BRI for predicting OAB was lower in men (5.151) compared with women (5.383), indicating that men were more susceptible to the change in BRI than women (Supplementary Table 2).

## Discussion

This study was novel in exploring the correlation between BRI and OAB through a cross-sectional analysis. The weighted multivariate logistic regression analyses showed that BRI remained an independent risk factor for OAB after controlling for all covariates. The RCS curve indicated no nonlinear dose–effect association between BRI and OAB. Furthermore, a higher BRI corresponded to a higher likelihood of developing OAB. BRI exhibited the highest AUC for predicting OAB compared with anthropometric measures such as BMI, WC, and weight. These findings indicated that BRI might be a valuable tool for anticipating the development of OAB, and addressing obesity could aid in preventing OAB.

Recent studies indicated a positive association between obesity and OAB. A study including 206 overweight women discovered that those with a body fat percentage (BFP) higher than 32% faced a 95% higher risk of OAB compared to those with a BFP of 32% or lower [28]. A prospective study by Alsannan et al. [29] in 2024 revealed that the risk of severe OAB was 5.8 times higher in overweight women and 18.6 times higher in obese women compared to women with a normal BMI. OAB patients had significantly higher BMI, weight, WC, and BRI compared with those without OAB in this study. Therefore, participating in physical activity to lower fat, particularly visceral fat, can aid in preventing OAB and reduce the likelihood of developing it, delivering additional benefits to the population.

In general, BMI is commonly used as a diagnostic tool for identifying obesity and can indicate body fat distribution [30]. Nevertheless, conventional anthropometric indicators have restrictions in differentiating between fat and muscle [31]. The BRI is an indicator of obesity formulated from height and WC, which provides a more comprehensive overview of body fat and visceral fat compared to other anthropometric indices like BMI [15, 32, 33]. According to a meta-analysis, BRI was found to be a more effective predictor of metabolic syndrome compared to waist-to-hip ratio, BMI, body adiposity index, and body shape index [34]. Additionally, BRI is also correlated with several diseases, including colorectal cancer [35], cardiovascular disease [36–38], and diabetes [39]. Nevertheless, the correlation between BRI and OAB remains unclear. The new findings of this research indicated a positive association between BRI and OAB and identified the optimal cutoff value for BRI as 5.124.

Developing an accurate predictor is crucial for the effective management of OAB. Both teams, led by Liu and Xu, proposed the BRI as an easy and affordable way to asses cardiometabolic risk among Chinese adults [37, 40]. A recent study discovered an association between higher baseline BRI and an increased risk of developing metabolic syndrome (MetS). Identifying patients with high baseline BRI may help predict those at risk for MetS [41]. Available research indicates that MetS possibly plays a significant role in the progression of OAB. OAB associated with MetS can be considered a specific subtype of OAB [10, 42]. Despite the lack of direct evidence linking BRI and OAB, a correlation may exist between MetS and OAB, explaining the increased likelihood of OAB in individuals with high BRI levels. This index exhibits superior predictive capabilities compared with other anthropometric indices. Compared with other biomarkers or prediction models, BRI was considered a simple, low-cost, noninvasive tool for predicting OAB in this study [43, 44].

The prevailing notion is that OAB predominantly affects women, although an epidemiological study has suggested that the prevalence of OAB is comparable among adult men and women residing in the community, with rates of 16.0% and 16.9%, respectively [2]. Most patients participating in the clinical trials for OAB are women, accounting for about 70% of participants. This has led to a lack of knowledge about effective treatment options specifically designed for men [45]. Furthermore, diagnosing and treating OAB in men can be difficult because the symptoms often overlap with those of benign prostatic hyperplasia [46]. This study was novel in revealing that men were more susceptible to the change in BRI for OAB compared with women. Moreover, the optimal threshold value of BRI for predicting OAB was lower in men (5.151) compared with women (5.383), suggesting that men might derive significant benefits from BRI in the early-stage prediction of OAB compared with women.

### Strengths and limitations

This study had the following strengths. First, it innovatively examined the association between BRI and OAB in the US population. Second, the current findings showed that the BRI was closely associated with OAB, and BRI, as a novel anthropometric method, was superior to traditional anthropometric methods in screening for OAB. Third, the findings of the different models and subgroup analyses after adjusting for confounders suggested a robust correlation between BRI and OAB. Finally, the study included a sizable population sample that might accurately represent the characteristics of the entire national population. Sampling weights were also taken into account in the analyses, thus improving the ability to make statistical inferences. Nonetheless, there were also some limitations. First, this research used a cross-sectional design and thus could not determine a causal relationship between the BRI and the prevalence of OAB. Second, all participants in this research were from America. Therefore, the results might not apply to other national populations. Third, the patient’s responses to OAB-related inquiries relied solely on self-recollection, without the diagnostic information from physicians and specialists. Finally, researchers could not determine whether certain potential confounders existed that were not addressed by the study.

## Conclusions

The current study demonstrated a significant correlation between elevated BRI and a higher likelihood of OAB, suggesting that BRI could be a practical tool for assessing OAB in clinical settings. Furthermore, effectively managing obesity may have a positive impact on the prevention of OAB.

### Electronic supplementary material

Below is the link to the electronic supplementary material.

Supplementary Material 1

## Data Availability

The datasets used and/or analyzed during the current study are available at https://www.cdc.gov/nchs/nhanes.

## Abbreviations

OAB: overactive bladder
BRI: body roundness index
RCS: restricted cubic spline
ROC: Receiver-operating characteristic
OR: odds ratio
CI: confidence interval
BMI: body mass index
WC: waist circumference
NHANES: National Health and Nutrition Examination Survey
UUI: urinary urgency incontinence
OABSS: Overactive Bladder Symptom Score
TC: total cholesterol
FBG: fasting blood glucose
LDL-C: low-density lipoprotein cholesterol
TG: triglycerides
PIR: poverty income ratio
AUC: area under the curve
BFP: body fat percentage
MetS: metabolic syndrome
