# Supplementary material for: Association between body roundness index and overactive bladder: results from the NHANES 2005–2018
Source: Lipids Health Dis. 2024 Jun 12;23:184. doi: 10.1186/s12944-024-02174-1 (PMC11167800; doi:10.1186/s12944-024-02174-1)
Supplement: Supplementary file 1 — Supplementary Material 1 [file 12944_2024_2174_MOESM1_ESM.docx]

**Supplementary Table 1. Association between BMI, WC and BMI and overactive bladder^a^.**

| **Characteristic** | **Model 1**  **OR (95%CI), P-value** | **Model 2**  **OR (95%CI), P-value** | **Model 3**  **OR (95%CI), P-value** |
| --- | --- | --- | --- |
| BMI | 1.05 (1.04,1.06), **<0.001** | 1.05 (1.04,1.06), **<0.001** | 1.05 (1.04,1.06), **<0.001** |
| Tertiles of BMI |  |  |  |
| Tertile1 | Reference | Reference | Reference |
| Tertile2 | 1.39 (1.20,1.61), **<0.001** | 1.25 (1.08,1.46), **0.004** | 1.26 (1.07,1.48), **0.006** |
| Tertile3 | 2.15 (1.84,2.50), **<0.001** | 1.95 (1.67,2.28), **<0.001** | 1.81 (1.53,2.14), **<0.001** |
| P for trend | **<0.001** | **<0.001** | **<0.001** |
| WC | 9.68 (6.97,13.44), **<0.001** | 8.56 (5.78,12.65), **<0.001** | 6.84 (4.61,10.16), **<0.001** |
| Tertiles of WC |  |  |  |
| Tertile1 | Reference | Reference | Reference |
| Tertile2 | 1.84 (1.56,2.17), **<0.001** | 1.55 (1.30,1.84), **<0.001** | 1.51 (1.26,1.80), **<0.001** |
| Tertile3 | 2.47 (2.13,2.85), **<0.001** | 2.09 (1.79,2.45), **<0.001** | 1.87 (1.59,2.19), **<0.001** |
| P for trend | **<0.001** | **<0.001** | **<0.001** |
| Weight | 1.01 (1.00,1.01), **<0.001** | 1.01 (1.01,1.02), **<0.001** | 1.01 (1.01,1.02), **<0.001** |
| Tertiles of Weight |  |  |  |
| Tertile1 | Reference | Reference | Reference |
| Tertile2 | 1.14 (0.99,1.32), 0.071 | 1.37 (1.17,1.59), **<0.001** | 1.33 (1.14,1.55), **<0.001** |
| Tertile3 | 1.32 (1.13,1.53), **<0.001** | 1.95 (1.65,2.31), **<0.001** | 1.79 (1.50,2.13), **<0.001** |
| P for trend | **<0.001** | **<0.001** | **<0.001** |

Abbreviations: PIR, family poverty income ratio; FBG, fasting blood glucose; TC, total cholesterol; TG, triglyceride; LDL-C, low-density lipoprotein cholesterol; BMI, body mass index; WC, waist circumstance; BRI: body roundness index.

^a^Model 1: unadjusted; Model 2: adjusted for age, gender, race, educational attainment, and marital status; Model 3: adjusted for age, gender, race, educational attainment, marital status, PIR, smoking status, drinking status, hypertension, diabetes, FBG, TC, TG, and LDL-C.

**Supplementary Table 2. Diagnostic efficacy of ROC analysis of obesity-related indices for overactive bladder.**

| **Subgroups** | **Anthropometric**  **Measures** | **Best**  **thresholds** | **Sensitivity** | **Specificity** | **Positive predictive value** | **Negative predictive value** | **AUC (95%CI)** | **P for difference**  **in AUC** |
| --- | --- | --- | --- | --- | --- | --- | --- | --- |
| **Male** |  |  |  |  |  |  |  |  |
|  | BRI | 5.151 | 0.588 | 0.402 | 0.223 | 0.881 | 0.608 - 0.645 | Reference |
|  | BMI | 30.775 | 0.394 | 0.277 | 0.218 | 0.859 | 0.541 - 0.581 | **<0.001** |
|  | WC | 1.042 | 0.519 | 0.357 | 0.222 | 0.872 | 0.588 - 0.627 | **<0.001** |
|  | Weight | 96.450 | 0.325 | 0.256 | 0.199 | 0.849 | 0.509 - 0.549 | **<0.001** |
| **Female** |  |  |  |  |  |  |  |  |
|  | BRI | 5.383 | 0.685 | 0.440 | 0.317 | 0.857 | 0.645 - 0.676 | Reference |
|  | BMI | 27.595 | 0.692 | 0.493 | 0.295 | 0.847 | 0.609 - 0.641 | **<0.001** |
|  | WC | 0.936 | 0.724 | 0.494 | 0.304 | 0.860 | 0.630 - 0.662 | **<0.001** |
|  | Weight | 73.250 | 0.597 | 0.455 | 0.281 | 0.820 | 0.579 - 0.612 | **<0.001** |

Abbreviations: PIR, family poverty income ratio; FBG, fasting blood glucose; TC, total cholesterol; TG, triglyceride; LDL-C, low-density lipoprotein cholesterol; BMI, body mass index; WC, waist circumstance; BRI: body roundness index.


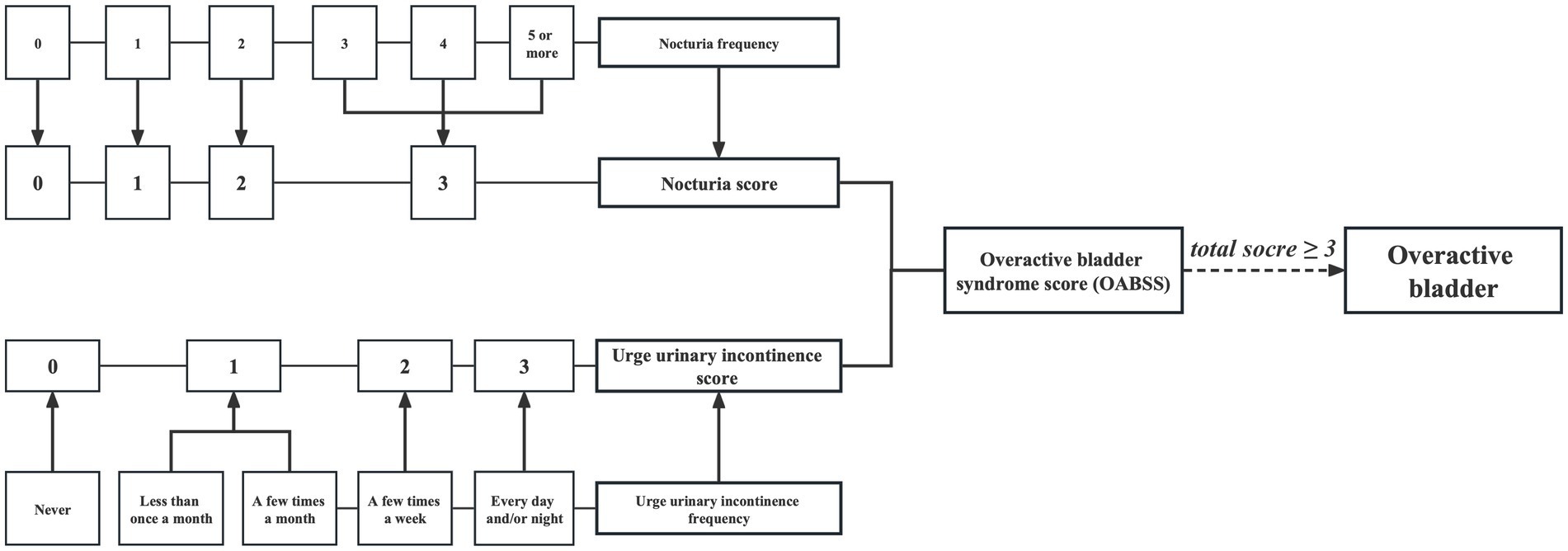


**Supplementary Figure 1.** Flow diagram of the overactive bladder diagnosis based on overactive bladder syndrome score.


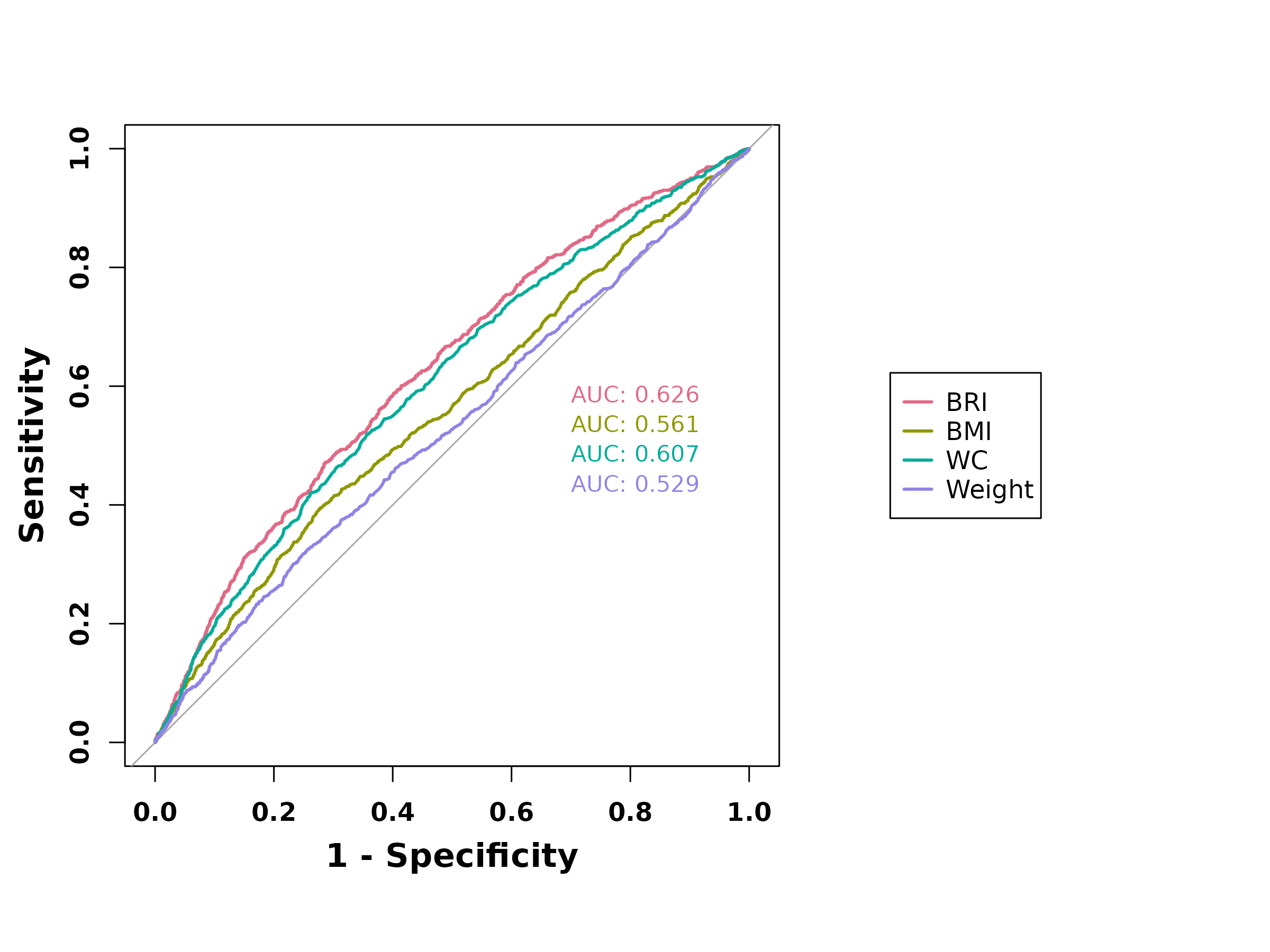


**Supplementary Figure 2.** Receiver operating characteristic (ROC) curve analysis for predicting overactive bladder in male. AUC: area under the curve; BRI: body roundness index; BMI: body mass index, WC: waist circumference.


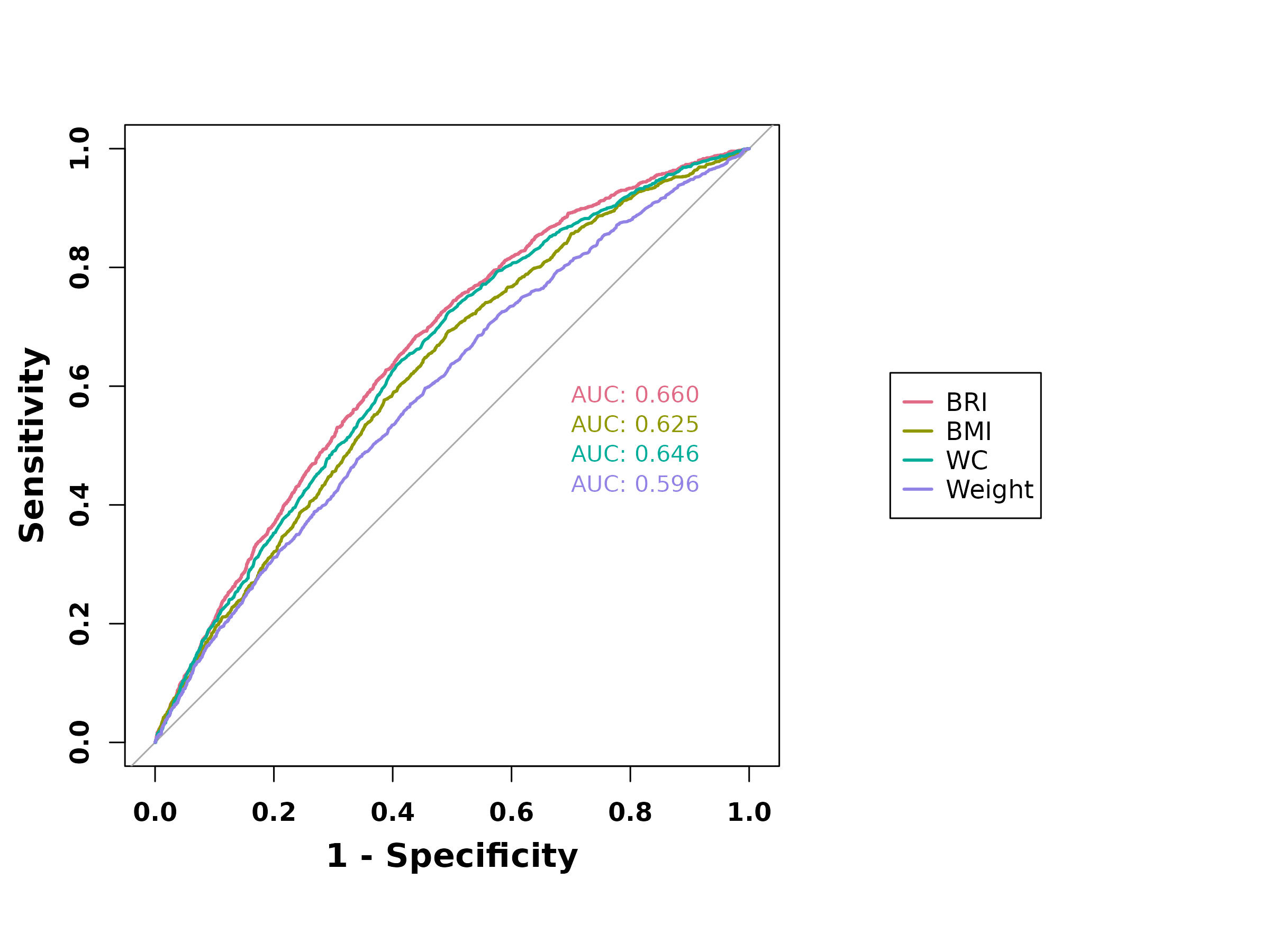


**Supplementary Figure 3.** Receiver operating characteristic (ROC) curve analysis for predicting overactive bladder in female. AUC: area under the curve; BRI: body roundness index; BMI: body mass index, WC: waist circumference.
